# Evidence of a pharmacodynamic EEG profile in rats following clonidine administration using a nonlinear analysis

**DOI:** 10.1186/1753-4631-5-4

**Published:** 2011-06-26

**Authors:** David-Olivier D Azulay, Benjamin Renoux, Magnus Ivarsson

**Affiliations:** 1 Pfizer Global Research and Development, Ramsgate Road, Sandwich, CT13 9NJ, UK; 2 Ecole des Mines d'Alès, Avenue Clavières, 30319, Alès, France

## Abstract

**Background:**

Changes caused by clonidine in rodent electroencephalograms (EEG) have been reported with some inconsistency. For this reason, a pre-clinical study was conducted in order to confirm previous findings with both a standard spectral analysis and a sleep stage scoring procedure. In addition, a nonlinear technique for analysing the time-varying signals was implemented to compare its performance against conventional approaches.

**Results:**

The nonlinear method succeeds in quantifying all dose-related responses from the data set relying solely on the EEG trace.

**Conclusions:**

Nonlinear approaches can deliver a suitable alternative to the sleep-stage scoring methods commonly used for drug effect detection.

## 1 Background

The role of the noradrenergic system in sleep physiology has been studied extensively using different pharmacological approaches [1-5]. It is well established that the non-selective alpha-2 agonist clonidine promotes non-rapid eye movement (NREM) sleep in both humans and rats mainly by activating presynaptic inhibitory autoreceptors and thereby inhibiting noradrenergic neurotransmission. However the specific changes caused by clonidine directly in the EEG is less clear both in humans and rats.

In humans, clonidine has been shown to cause changes in the EEG [6-8]. have noticed that power is increased in the 1.0-4.0 Hz range while decreased in the 8.0-12 Hz range. Recently published data suggest that clonidine causes different effects on the EEG depending on level of exposure [9]. In this study the authors showed a significant decrease in power in the 0.5-12 Hz band during rapid eye movement (REM) sleep only.

The findings with clonidine from rodent EEG studies are equally ambiguous [10]. showed that clonidine caused the power to decrease in the 0.1-4 Hz range and increase in the 4.1-8 Hz range compared to vehicle controls specifically in the NREM sleep stage. In contrast, [11] showed that the changes caused by clonidine were characterized by a significant increase in nearly all the frequency range from 1-30 Hz, with a peak at 13 Hz. Whether these differences in the reported pre-clinical and clinical findings with clonidine are only due to varying exposure levels or whether there are other additional causes (e.g. definitions of bands in the human and rodent studies, varying electrode derivations, changes restricted to specific sleep stages) is unclear.

An EEG recording is the superposition of a number of electrical signals eminating from various regions in the brain. If all these activities generated perfect superpositions of electrical sine wave oscillations then a linear method like a Fourier transform would expose these periodic components [12]. In the case of non-regular time-varying signals, the task is considerably more complex and requires several steps. Nonlinear approaches aim first at detecting if a deterministic structure exists in the waveform before any further calculations. The presence or absence of determinism in a given data set is the corner stone of its numerical analysis since it completely defines which category of algorithms is practicable.

In terms of system dynamics, time series EEGs can be viewed as 2-dimensional windows open to a *m*-dimensional state space where time points repeat complex cyclic patterns [13]. Since a reliable approximation of *m *would help understand the underlying mechanisms involved, algorithms designed to estimate this dimension have been applied [14-16]. Although none of the tested algorithms was able to deliver a definitive answer so far, these numerical methods are capable of extracting useful parameters for very specific phases like sleep [17-19], epileptic [20], schizophrenic [21] or anaesthesia [22] stages. These examples suggest that the measurement of the EEG determistic aspect can quantify different episodes of brain activities by using nonlinear methods.

The aim of this study was to identify a sensitive non-linear model of EEG analysis that can extract the pharmacodynamic signal of clonidine from EEG recordings from freely moving animals.

## 2 Materials and methods

All animal experiments were carried out in accordance with the United Kingdom Animals (Scientific Procedures) Act 1986 and associated guidelines and approved by the local ethics committee. Rats were implanted with radiotelemetry transmitters (Data Sciences International, St Paul, MN, USA) intraperitoneally under isoflurane anaesthesia for the recording of the EEG and electromyogram (EMG). The cortical EEG electrodes (stainless steel screw electrodes) were implanted epidurally over the left parietal cortex (2.0 mm anterior and 2.0 mm lateral to lambda) and over the left frontal cortex (2.0 mm anterior and 2.0 mm lateral to bregma) for a fronto-parietal EEG recording [23-25]. The electrodes and leads attached to the skull were covered with dental acrylic and a second pair of electrodes was attached to the neck muscles to measure general EMG activity needed for the sleep stage analysis only. The rats were allowed to recover from the implantation of the device for at least 2 weeks, and the experiment was started once the animals were certified fit to continue by a veterinary surgeon. Animals were singly housed on a standard 12-12 h light-dark cycle and received standard diet and water ad libitum.

EEG and EMG data were continuously sampled at 250 Hz and the spectral upper limit was set at 40 Hz, with Data Sciences International hardware and software for 12 hours immediately following administration of drug at light onset. Animals were orally dosed with 0.03 (low dose (LD)), 0.1 (medium dose (MD)) or 0.3 (high dose (HD)) mg/kg clonidine or vehicle (VC) (0.5% w/v methylcellulose + 0.1% v/v Tween 80) at light on-set in a four way cross-over design with at least 48 hours between each dose. Recording of EEG and EMG signals began immediately after dosing. In addition to the sampling of these two signals, a general activity measure and core body temperature were collected in parallel. Due to an archiving issue, not all the files were available for the analysis.

Besides the nonlinear technique detailed below, a sleep stage analysis was completed to identify potential correlations between both outputs. A sleep stage discriminator was programmed whose logic is similar to a visual analysis [24]: its algorithm is summarised in Table 1.

**Table 1:** Sleep stage scoring principle

mod	low EEG	high EEG
low EMG	REM	NREM
high EMG	WAKE	WAKE

The modulus (mod) is defined as the absolute value of the area under the curve from the EEG or EMG traces.

EEG traces were cut into 16.384 second epochs (2^12 ^points). Since one epoch of EEG data values tend to follow a gaussian distribution the Kolmogorov-Smirnov test was used to filter artefacts [26]: 2% of the epochs were rejected from our data set. The integral local deformation (ILD) time-embedding window algorithm was implemented to evaluate the deterministic structure of the data [27]; in this work a validation was carried out against known systems [28].

**Figure 1:**
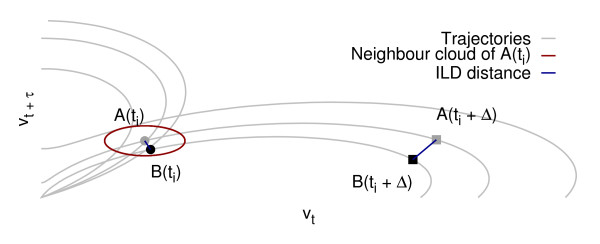
**Illustration of the ILD measurement in 2 dimensions**. The distance between points A and B is computed at different times and its variation used to estimate the overall deviation.

For a given dimension *m *and time delay *τ*, the signal is represented by points **x**(*t*) = [*v_t_*, *v*_*t*+*τ *_, ... *v*_*t*+(*m*-1)*τ*_]*^T ^*whose displacements are tracked and quantified according to the homogeneity of their flow. If some unknown but deterministic effects drive the data, they should produce a small perturbation to a moving cloud of points as time passes by: points on neighbouring trajectories remain neighbouring points for small evolution times Δ as sketched in Figure 1[27]. The ILD algorithm calculates the average deviation in terms of distance between points in a cloud for a given (*m*, *τ*) couple (up to a normalisation factor).

To allow comparisons with published results, the data analysis relied on a 0-40 Hz power spectrum to identify drug-related signals in the raw EEG data. Efforts were concentrated in distinguishing a clear effect in the EEG down to the lowest administered dose of clonidine.

## 3 Results

**Figure 2:**
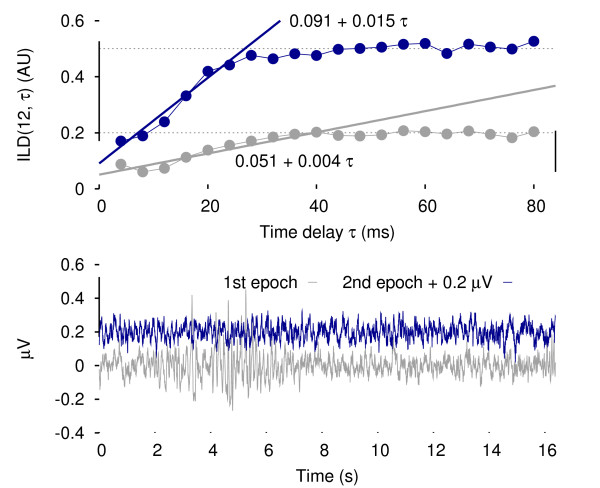
**Results of the ILD calculations on two different epochs from rat 1/VC**. Since a high plateau is reached faster than a low one, the slope b correlates with the level of the plateau.

The ILD procedure was run on every EEG epoch for each dimension *m *∈ [2,12] and time delay *τ *∈ [4,80] in ms (in 4 ms increment) as input parameters. Plots identical to Figure 2 were generated to perform visual verification; from these data two groups were identified:

1. where the plateau of the graph is lying high around 0.5.

2. where the plateau of the graph is lying low around 0.2.

From a time-embedding perspective there is no local minimum, that is to say no time delay *τ *which induces a minimal deviation. The ILD curves systematically reach a plateau after a few iterations. The dimension was arbitrarily fixed at *m *= 12 since all the plots produced with a dimension estimation *m *greater than 10 had their plateaux converging.

**Figure 3:**
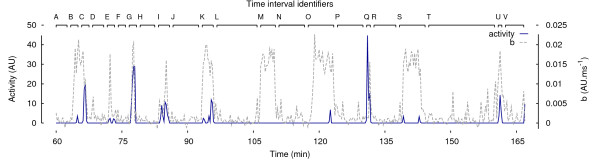
**Activity and ***b ***overlap for rat 1/VC**. Some recurring patterns are visible throughout the time course.

Nevertheless these plots provide a quantification of the structure of the signal and details are presented on how the steepness of the slope preceding the plateau is a valuable measurement in terms of characterisation of the time series. The ascending part can be modelled with a simple linear *a *+ *bτ *fit of the first 6 points. The value of *b *is therefore a measure of both the rate of the convergence of the ILD computations and the height of the plateau. If this nonlinear derived parameter *b *is overlapped with the activity channel to investigate correlations, as shown in Figure 3, four distinct (activity, *b*) combinations are possible:

1. (null activity, high *b*) periods are regular throughout the experiment (e.g. time intervals B, M, O, S). They explain most of the main wide peaks in the plot.

2. (null activity, low *b*) periods are distributed over the time course (e.g. time intervals A, D, F, H, J, L, N, P, R, T, V). They cause the recurrent flat parts in the plot.

3. (non-null activity, high *b*) periods are quite short and randomly localized (e.g. time intervals C, E, G, I, K, Q, U), generating rather sharp peaks.

4. (non-null activity, low *b*) periods are quasi inexistent in the experiments.

**Figure 4:**
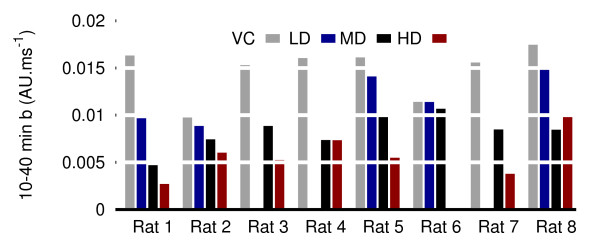
**Inverse dose-response signal obtained with the slope parameter ***b ***within the 10-40 minute window**.

To demonstrate the potential of *b *at this early part of the analysis, a 30-minute window (from 10 to 40 minutes) displays in all rats an average value that already produces an inverse dose-response relationship (see Figure 4). This time frame is consistent with the pharmacokinetic properties of clonidine reported by [29,30]: clonidine dosed at 0.25 mg/kg peaks in the brain within 2 min and disappears at an average half-life rate of 70 min.

**Figure 5:**
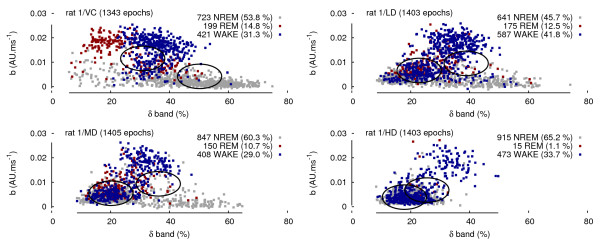
**Instances of (***δ***, ***b***) plots during the first 6 hours and their correlations with sleep/wake stages**. The centers of the fuzzy clusters coincides with the centers of the ellipses. The cluster density is an arbitrary measure of the number of points contained within an ellipse. The persistence of the REM sleep inhibition reported by [10] is noticeable.

The discriminant function of *b *is improved by pairing it with the normalised power of the *δ *band, defined as the ratio of the power of the 0.5-4 Hz band to the 0-40 Hz band from a Fourier transform based spectrum. Using *δ *as the abscissa and *b *as the ordinate for each epoch, vehicle plots display two clusters (WAKE/REM vs NREM) whilst drug plots display mainly a diffused cluster during the first six hours of the experiment as seen Figure 5.

**Figure 6:**
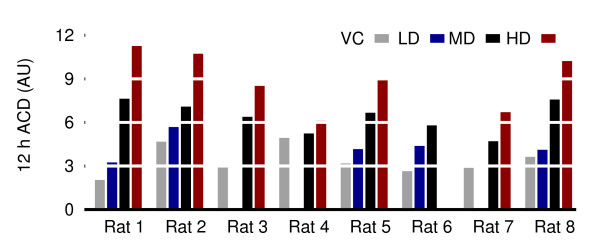
**Average of the ACD for the first 12 hours**. A clear dose-response signal is visible. The individual cluster densities over time are all plotted in Figure 7.

**Figure 7:**
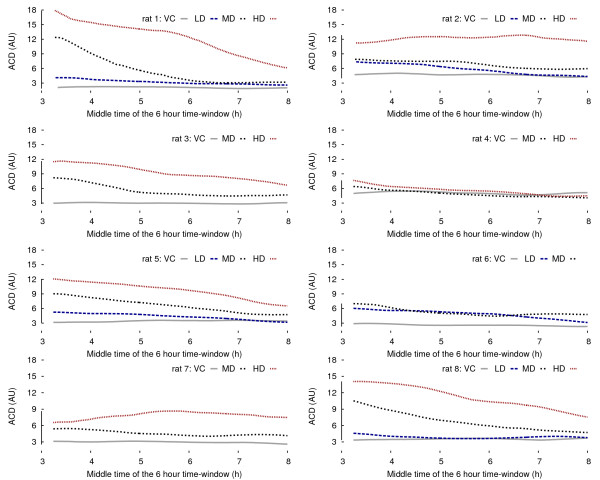
**Individual pharmacodynamic profiles obtained from the ACD time courses**. The order of the escalating doses is maintained during the different experiments.

These two-dimensional patterns were quantified using a fuzzy *k*-means clustering algorithm [31] taking into account the following assumption: a 2 cluster input data set, expected for the baseline condition, is bound to produce an outcome very different from a 1 cluster input data set, expected for a drug effect, if fed into a 2 cluster search. Each point is given a belonging probability proportional to the inverse of its distance from the center of a cluster. A clear-cut cluster gathers points with high probabilities, that is to say close to its center, whereas a diffused one encompasses points further away. Hence, the quality of the output clustering is the measure of interest: the area covered by the two clusters becomes smaller when the amount of drug increases as seen in Figure 6, but the total number of points remains approximately the same. The density of the clusters is then greater with a drug onboard. This is the reason why the average cluster density (ACD) criterium renders the clear dose-response relationship observed in Figure 7.

**Figure 8:**
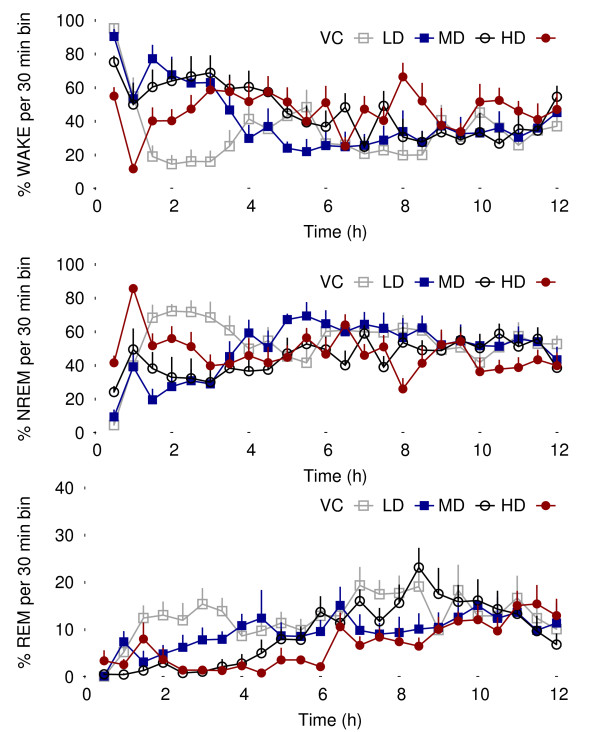
**Sleep stage distributions**. The NREM "rebound decrease" reported by [10] is visible during the first hour. Nevertheless a significant difference is only observed for the highest dose (see Table 2).

Finally, the level of confidence in the (*δ*, *b*) couple is raised to a higher degree if the response exhibits a pharmacodynamic time response. A 6 hour sliding time window reveals that the proposed measurement is quite suitable as a pharmacological biomarker: Figure 7 shows the evolution of the ACD over 12 hours for all 8 rats. Since time is now a proper dimension, the curves can be interpreted as pharmacodynamic profiles: the stacking order of the escalated doses is preserved and the drug effect disappears progressively with time. For the sake of comparison, sleep stage distributions shown in Figure 8 are more difficult to interpret.

## 4 Discussion

A common technique to detect drug impacts on EEGs relies on sleep stage scores [32]: time proportions of sleep stage periods [24] or powers per bands [23,32] have demonstrated pharmacological effects. Ideally, from a cost and time perspective, the pharmacodynamic effect of a novel drug should be assessed in parallel to standard first in human (FIH) trials. This could potentially be done with a sensitive automatic analysis based on standard wake EEG recordings. However clinical sleep architecture studies tend to be more expensive, mainly due to the time it takes to run these studies in specialist sleep centres. The detection of a pharmacological response directly from the EEG trace would therefore be the preferred solution in an early clinical trial, if achievable.

Nevertheless, links to sleep stages are detailed here for comparison purposes but not retained as part of the final nonlinear analysis workflow. The normalised powers of the different bands per sleep-wake stage during the control condition also confirm the classic distribution of the different frequency bands. For the vehicle dose, a high power in the *δ *band is associated with the occurrence of NREM sleep stages and a *θ *band power dominates in REM sleep from a fronto-parietal electrode configuration in rodents. The vehicle plot in Figure 5 shows that NREM epochs are synchronised with a high *δ *and low *b *values (bottom right) whereas REM epochs display lower *δ *but higher *b *values (top left). One could potentially rely of the localisation of each point in the different regions of the graph to perform an a posteriori sleep stage epoch scoring.

Clonidine has been shown to reduce the firing of noradrenergic neurons in the Locus Coeruleus (LC) via autoreceptors, thus altering the input to the cortex and causing a change in the texture of the recorded EEG signal, which might be reflected in the non-linear parameter *b *[10,33].

Table 2 supports the findings described by [10] that the administration of clonidine caused an increase in NREM sleep and a dose dependent decrease in *δ *power specifically in NREM sleep rather than a significant increase of the total power as described by [11]. One possible explanation for the discrepancy between the different studies is that [11] recorded the effect of clonidine locally in the prefrontal cortex, while both the outcomes described in this study and those by [10] were carried out with a similar fronto-parietal/occipital electrode configuration.

**Table 2:** Averaged normalised powers in spectral bands for the distinct doses and sleep-wake stages during the first hour

	NREM powers in %					REM powers in %					WAKE powers in %				
	*δ*	*θ*	*α*	*β*	%/h	*δ*	*θ*	*α*	*β*	%/h	*δ*	*θ*	*α*	*β*	%/h
VC	**33.2**	26.3	16.2	14.4	28.5	20.4	**37.8**	13.8	11.7	4.3	**31.2**	28.3	11.8	11.5	67.2
LD	**30.2**	28.7	16.4	14.9	27.5	25.9	**30.8**	13.0	14.1	3.5	28.3	**30.7**	12.2	12.4	69.0
MD	29.6	**30.3**	15.8	15.0	39.1	26.8	**29.1**	13.3	15.2	0.7	27.1	**32.1**	12.6	13.4	60.2
HD	27.6	**33.6**	15.8	14.7	65.7^†^	25.7	**31.5**	13.6	15.1	4.1	26.3	**33.6**	13.6	14.2	30.2^†^

Powers in bold indicate the maximum of the four band powers (*δ *= 0.5-4 Hz, *θ *= 4-8 Hz, *α *= 8-12 Hz and β = 12-20 Hz) for each stage and dose. The percentage of time spent during the first hour (%/h) per stage and dose is reported as well. Significant difference (*p *< 0.05) between VC and HD (comparison made using a one-way ANOVA) is marked with the † symbol.

Given a new drug, there is always a possibility that its unknown effect might corrupt the usual sleep stage classifiers that are well defined for healthy subjects, but less so for preclinical sleep scoring; as an example of changes in the EEG signal irrespective of sleep stage see [34].

Assumptions solely formed on sleep scores would then be inconclusive. Continuous characterisations like the nonlinear one presented above are likely to be more robust in these particular cases.

This work fully supports the idea that nonlinear techniques are valuable solutions for analysing EEG. The results presented here are consistent as clonidine is known to affect sleep [35] and nonlinear approaches are known to discern sleep stages [28]. The combination with the standard *δ *band commonly associated with the awake stage reinforces the plausibility of having extracted a genuine signal from this data set.

In conclusion, this study describes a generic mathematical framework that can extract a pharmacodynamic profile of clonidine from raw EEG data collected from a fronto-parietal electrode derivation in freely-moving rodents, which could offer an alternative approach to study drug effects in early clinical trials. The model is based on a numerical analysis tool whose field of application is nonlinear dynamic systems. This innovative approach can potentially provide a translatable analysis methodology for assessing central pharmacodynamic effect and bridge the preclinical and clinical EEG observations.

## Competing interests

DOA and MI receive salaries from Pfizer.

## Authors' contributions

DOA and BR have developed and implemented the algorithms used in the analysis. MI conducted the rat experiments. DOA and MI helped to draft the manuscript. All authors read and approved the final manuscript.
